# AMPK/mTORC2/AKT-473/RUNX2 signaling axis modulates epithelial-mesenchymal transition and bone tropism in breast cancer

**DOI:** 10.3389/fonc.2026.1785903

**Published:** 2026-04-10

**Authors:** Meher Bolisetti Gayatri, Abhayananda Behera, Suresh Chava, Preeti Vyakaranam, Ganesh Venkatraman, Suresh Kumar Rayala, Aramati Bindu Madhava Reddy

**Affiliations:** 1 Department of Animal Biology, School of Life Sciences, University of Hyderabad, Hyderabad, India; 2 Department of Bio-Medical Sciences, School of Bio Sciences & Technology, Vellore Institute of Technology, Vellore, India; 3 Department of Biotechnology, Indian Institute of Technology Madras, Chennai, India

**Keywords:** EMT metformin, metastasis, mTORC2, P-AMPK, RUNX2

## Abstract

**Introduction:**

Metformin, a widely used biguanide for the treatment of type 2 diabetes, has been extensively studied for its potential anti-cancer properties, primarily attributed to its inhibitory effects on mTORC1 signaling. However, accumulating evidence suggests that its impact on tumor progression is highly context-dependent, varying with cellular and metabolic conditions. In this study, we investigated the mechanistic effects of metformin on RUNX2 and mTORC2 signaling pathways in breast cancer.

**Methods:**

The study was conducted using in silico, in vitro, and in vivo analysis. In vitro experiments was carried out using MDA-MB-231 breast cancer cells to evaluate the regulatory effects of metformin on RUNX2 and mTORC2 signaling. Gene knockdown approaches targeting RICTOR were employed to assess pathway interactions, and molecular analyses were performed to examine the involvement of AMPK and GSK3β in regulating RUNX2 stability and downstream signaling events. Tumor samples were analyzed to validate the clinical relevance of the observed molecular alterations. Additionally, in vivo studies were performed to assess the functional impact of the identified signaling axis on tumor progression and metastatic potential.

**Results:**

Metformin treatment resulted in enhanced mTORC2 activity in a RUNX2-dependent manner, mediated through AMPK-driven stabilization of RUNX2. Furthermore, silencing of RICTOR, a critical component of mTORC2, induced RUNX2 degradation via a GSK3β-dependent mechanism, indicating a reciprocal regulatory relationship between RUNX2 and mTORC2 pathways. Functional analyses demonstrated that the AMPK–RUNX2–mTORC2 signaling axis promotes epithelial–mesenchymal transition (EMT) and enhances the bone metastatic potential of breast cancer cells.

**Discussion:**

These findings reveal a context-dependent role of metformin in modulating metastatic signaling pathways through the AMPK–RUNX2–mTORC2 axis. The study highlights the complexity of metformin’s action in biology and underscores its potential to differentially regulate tumor progression and metastasis depending on the molecular context.

## Graphical Abstract

**Figure:**
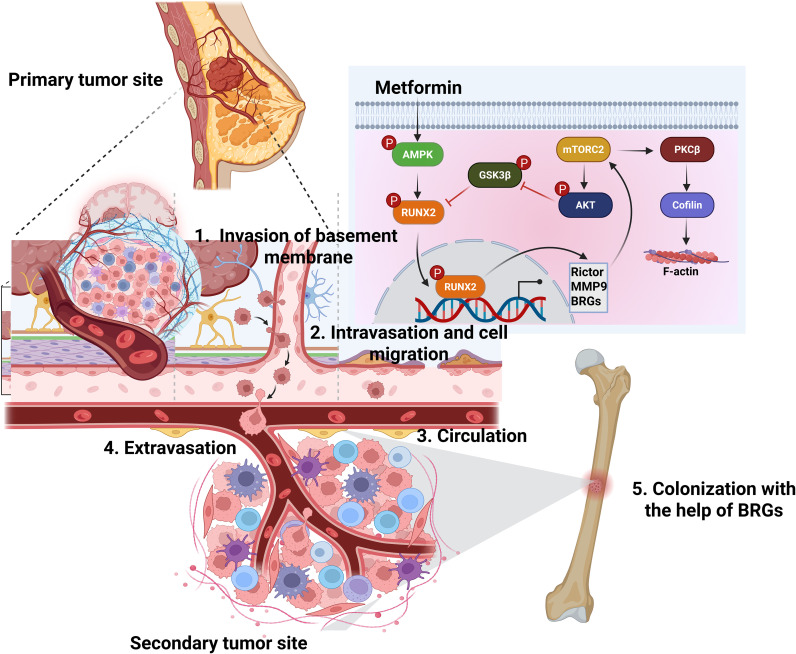
Schematic representation of metformin-mediated stabilization of mTORC2/RUNX2 axis. This figure summarizes, along with the findings from the current study, where, at physiological levels, activation of mTORC2 by PIP2 resulted in activation of AKT by phosphorylation at Ser473. pSer473AKT inhibits GSK3β by phosphorylation at Ser9. Inhibitory/in-active Ser-9 GSK3β fails to phosphorylates RUNX2, which otherwise would be primed to ubiquitination. Metformin mediated activation of AMPK results in phosphorylation of RUNX2 at Ser 118 position, resulting in enhanced transcriptional activity of RUNX2, which transcribes for RICTOR, MMP-9 and VEGF. The downstream targets of RUNX2 act to promote EMT and bone metastasis of breast cancer cells. Hyperactivate mTORC1 inhibits mTORC2. Inhibition of mTORC2 results in destabilization of RUNX2 through the loss of pSer473 AKT, which leads to activation of GSK3β. Active GSK3β interacts with RUNX2 and primes it for ubiquitination.

## Introduction

Breast cancer, a global health challenge and the second most prevalent cancer, remains the major cause of death worldwide (1). RUNX2 (Runt-related transcription factor 2), a bone morphogenic factor, is highly expressed in breast cancer (2), particularly in aggressive tumor subtypes (3). RUNX2 is one of the major transcription factors involved in bone development (4). RUNX2 has a vast target repertoire and affects several pathways, including but not limited to proliferation, cell cycle progression, DNA damage response, which play a key role in cancer transformation (5). Notably, the expression of RUNX2 endows breast cancer cells with an osteoblast like phenotype by upregulation of bone specific proteins like osteopontin (6), osteonectin (7). This molecular alteration facilitates the homing, colonization and survival of breast cancer cells in the bone, causing osteolytic lesions and initiation of secondary tumors (8–10). Furthermore, ectopic expression of RUNX2 confers drug resistance and enhances metastatic ability, making RUNX2 a potent druggable target for cancer treatment (10–12), Despite its significance, the intricate molecular players underlying these processes remained elusive now.

Metformin, a widely used anti-glycemic drug, has gained attention as a potent anti-cancer agent. It exerts its effects by activating AMP-activated kinase (AMPK), a crucial cellular energy sensor (13). AMPK regulates energy homeostasis by inhibiting pathways involved in energy consumption (like cap-dependent protein synthesis (14)) and gluconeogenesis (15). Preclinical studies have demonstrated that metformin can inhibit cell proliferation and tumor growth through the AMPK/P53 axis (16–19). However, clinical trials investigating metformin’s anti-cancer ability in solid tumors, especially in breast cancer (20–22), have not yielded promising outcomes. The molecular mechanisms underlying this failure remain unclear, however. Bone metastasis is the most common anomaly associated with breast cancer pathogenesis (23). Most of these cells express osteoblast-like genes to metastasize and home in bone (24). The bone microenvironment not only provides these cells with the growth factors required to support their proliferation, but also contributes to the development of drug resistance (25). Despite being a potent therapeutic candidate for cancer treatment (26), the effect of metformin on bone metastasis of breast cancer cells remains poorly understood. Recent studies have shown that RUNX2 (27, 28) as well as mTORC2 (Mechanistic target of rapamycin complex 2) (29) play a key role in breast cancer metastasis.

mTORC2is a crucial regulator of cellular metabolism, cytoskeletal organization, and survival. Unlike mTORC1 (Mechanistic target of rapamycin complex 1), which primarily governs protein synthesis and cell growth, mTORC2 plays a pivotal role in maintaining metabolic homeostasis by phosphorylating AGC kinases, a group of proteins including AKT, SGK1, and PKC (30, 31). Emerging evidence from our studies and others suggests a complex interplay between mTORC2, RUNX2, and AMPK in cellular differentiation and energy regulation (32–34). mTORC2 can activate AKT, which subsequently inhibits FOXO (Forkhead box O) transcription factors, modulating RUNX2 expression and osteogenic differentiation (34, 35). Additionally, AMPK, a key energy sensor, can indirectly suppress mTORC2 activity under low-energy conditions, thus influencing RUNX2-mediated osteogenesis (36). These regulatory interactions highlight mTORC2 as a mediator between cellular energy status and osteogenic differentiation.

The mTORC2 complex plays a pivotal role in regulating cytoskeletal organization, cell survival, and metabolic signaling through downstream kinases such as AKT and GSK3β (Glycogen synthase kinase-3β) (37). Activation of mTORC2 leads to the phosphorylation and inactivation of GSK3β, thereby preventing the proteasomal degradation of several transcription factors, including RUNX2 (32). RUNX2, a master regulator of osteogenesis and a known promoter of metastasis in cancer, is negatively regulated by active GSK3β-mediated phosphorylation, which targets it for ubiquitin-dependent degradation (38). Thus, mTORC2-mediated inhibition of GSK3β enhances RUNX2 stability and transcriptional activity, linking anabolic signaling pathways with metastatic gene expression.

Our recent work on RUNX2 regulation in bone and adipogenic development revealed that RUNX2 is a novel substrate of AMPK, an immediate effector molecule of metformin. Additionally, we observed that AMPK-mediated phosphorylation of RUNX2 increased its transcriptional activity (39). Given RUNX2’s protective role in metformin-induced osteogenesis, we aimed to investigate the dynamics of this interaction in breast cancer progression and metastasis. Our study shows that metformin treatment stabilizes the AMPK/RUNX2 axis in breast cancer cells, leading to mTORC2 upregulation. Consequently, breast cancer cells acquire osteocyte-like features, leading to the activation of MMP-9 and VEGF, which are involved in metastasis, thus promoting bone metastasis of breast cancer cells.

## Materials and methods

### Cell culture and chemicals

MDA-MB-231 (RRID: CVCL_0062) cells were grown in L-15 medium (Gibco, USA) supplemented with 2mM Glutamine, 15%FBS (Gibco, USA) and 1% Pen-Strep (Gibco, USA); MCF-7 (RRID: CVCL_0031) cells were grown in RPMI-1640 medium (Gibco, USA), HEK293T (RRID: CVCL_0063) cells were grown in DMEM (Gibco, USA) with 10% FBS (Gibco, USA) and 1% Pen-Strep (Gibco, USA). Cells were maintained at standard humidified conditions with 5% CO2 at 37 ^0^C. All cell lines were obtained from the American Type Culture Collection (ATCC, Manassas, VA, USA) and maintained according to ATCC-recommended protocols. Experiments involving MCF-7 and MDA-MB-231 cell lines were conducted with cells at fewer than 15 passages. Cell line authentication was verified through short tandem-repeat (STR) DNA profiling, in accordance with ATCC guidelines. Routine mycoplasma testing using isothermal PCR confirmed that all experiments were performed with mycoplasma-free cells. Metformin, dorsomorphin (compound C), MG-132 and LiCl were procured from Sigma, USA. Blasticidine S HCL was procured from Thermo Fisher Scientific.

### Sub-cloning

RUNX2 cDNA (NM_001024630.3) was procured from Genecopoeia, USA. RUNX2 site directed mutants S118A, D along with WT were subcloned into pDsRed1-N1 (Clonetech, USA) containing red fluorescent protein. The list of the primers used for site-directed mutagenesis is given in **Supplementary Table 1**. RUNX2 WT was subcloned into pBABE Puro vector followed by S118A and S118D site-directed mutation using the NEB site-directed mutagenesis kit (#E0554S).

### Nuclear and cytosolic extraction

Cells were treated with the indicated drugs for 6 hours then collected in 1X PBS. Subsequently, they were resuspended in buffer A (20mM Tris-HCl, pH-7.4, 10mM NaCl, 3mM MgCl2) (Sigma, USA) for 15 minutes on ice. After addition of 10% NP-40, the cells were vortexed at high speed, and then centrifuged for 10 minutes at 8,000 rpm in 4°C. The resulting supernatant represents cytosolic fraction. Buffer B (10mM Tris- HCl, pH-7.4, 2mM Na3VO4,0.5 mM DTT, 100mM NaCl, 1% Triton- X-100, 1mM EDTA, 5% glycerol, 0.1% SDS, 1mM NaF, 1mM EGTA, 0.5% deoxycholate) (Sigma, USA) was added to the pellet and the samples were incubated on ice for 30 minutes, followed by vortexing at 5 minutes intervals. Subsequently, the samples were centrifuged at 14,000 rpm for 20 minutes. The supernatant represents the nuclear fraction.

### Electrophoretic mobility shift assay

Cells were treated with the indicated drugs and nuclear extracts were prepared as mentioned above. The RUNX2 binding consensus on the RICTOR promoter served as a wild type probe (5’ TTAGGTACCACAGACATG 3’) and the mutant probe (5’ TTAGGTATTACAGACATG 3’) was designed with mutations at critical sites. Both wild type and mutant probes were labelled with γ-^32^P using the T4PNK (NEB, USA) enzyme following the manufacturer’s instructions. The labelled probe was then purified using G50 Spin columns (Sigma, USA) following protocol as mentioned previously (39).

### Immunoblotting and immunoprecipitation

Soon after the respective treatments, cells were lysed in 1X RIPA supplemented with protease and phosphatase inhibitor cocktails (Sigma, USA). IP was performed as described earlier (18) using 1 μg of anti-RUNX2 or anti-p-AMPK antibodies (Cell signaling technologies (CST), USA) at 4°C overnight. Protein A/G Plus- agarose beads (2.5mg/sample) (Santa Cruz, USA) were used to pull down the immunoprecipitated protein complexes and latter were subjected to immunoblotting. Antibodies used for immunoblotting were RUNX2, p-AMPK, p-AMPK substrate motif-specific antibody, p-P70S6K, P70S6K, RICTOR, RAPTOR, p-Akt (Ser 473), cofilin, p-cofilin (Ser 3), paxillin. All secondary antibodies were procured from CST, USA; MMP-9, VEGF, PKC, pan-Akt, ubiquitin, GSK3α/β, CDH11, AMPK and p-PKCβ were procured from Santa Cruz, USA. Lamin B1, p-GSK3β (Ser 9) and E-cadherin were procured form Abcam, USA. The signal was detected by Clarity Western ECL blotting substrates (Bio-Rad, USA) and images were processed using Bio-Rad Chemidoc MP system.

### Confocal analysis

Cells were grown to confluency (80%) on coverslips followed by treatment for indicated timepoints and then were washed with PBS before fixing in 4% formalin for 10 min at room temperature. Cells were stained for respective primary antibodies followed by respective fluorescence secondary antibodies as indicated. Images were captured using laser scanning confocal microscope (LSM 780, Carl Zesis).

### RT-PCR analysis

Total RNA isolation was carried out by TRIzol (Thermo Fisher Scientific, USA) method following protocol as described previously (40). The sequence of primers used along with their Tm is given in **Supplementary Table 2**. ΔΔCT method was used to quantify real-time data.

### Plasmid transfection

MCF-7 cells were seeded in six-well plate and grown to 50% confluency. 2μg of purified plasmid (RUNX2 WT or S118A or S118D) and GFP-luc were transfected using Lipofectamine-3000 (Thermo Fischer, USA) by following the manufacturer’s instructions. The cells were grown in OPTI-MEM for six hours, after which the medium was replaced with complete medium and grown for 48 hours (41).

### siRNA transfection

The siRNA for *RICTOR* (SI05109048), *RAPTOR* (SI00698677) and *RUNX2* (Hs.535845) were purchased from Qiagen (Netherlands) and Thermo fisher scientific (USA) respectively. 1µg of siRNA was transfected using RNAifect (Qiagen, Netherlands) following the manufacturer’s instructions. Cells were replenished with fresh regular medium after 6 hours of transfection.

### Cell sorting, retroviral transduction and infection

GFP-tagged MCF-7 cells were transfected and cultured until reaching confluence. Subsequently, the cells were trypsinized, washed twice with PBS, and resuspended in PBS containing 1% FBS. The cell suspension was kept on ice and subjected to cell sorting using a BD FACS AriaIII cell sorter. For the generation of retroviral particles, EV, RUNX2 WT, S118A, and S118D mutant plasmids were co-transfected into HEK-293T cells along with the pCEL-Eco vector. The filtered viral particles were then diluted with culture medium and transduced into GFP-tagged MCF-7 cells, along with the respective RUNX2 WT, S118A, and S118D mutants. Polybrene at a concentration of 8μg/ml (Sigma) was used during transduction, and the cells were incubated for 48 hours. Following infection, the medium was replaced with complete medium, and the cells were selected with blasticidin (2μg/ml, Invitrogen) and puromycin (2μg/ml, Sigma) for a period of 7 days.

### Trans-well migration assay

MCF-7 cells were seeded in a six well plate and transfected with RUNX2 WT, S118A, and D, followed by treatment with or without metformin (10mM) for 6 hours post-transfection. After transfection, cells were trypsinized and approximately 2×10^5^ cells per well were diluted in serum-free medium and added to the upper chamber of a transwell plate. The insert was coated with 100μL type I collagen (Thermo fisher scientific, USA) dissolved in 0.1% acetic acid (Finar, India) and left at 37 °C 2 hours for solidification. The insert was then washed twice in 1X PBS after removing excess collagen. 24 hours prior to this step, HEK-293T cells or U2OS cells were seeded in the lower chamber of the transwell plate in DMEM medium supplemented with 10% FBS and 1% pen-strep. The set up was then placed in the incubator at 37 °C for 16 hours. Following incubation, the medium from the inserts was aspirated and the cells were washed twice with 1X PBS followed by fixation in 3.7% formaldehyde (HiMedia, India) (dissolved in PBS) for 5 minutes, then washed in PBS. After fixation, cells were permeabilized with 100% methanol (Sigma, USA) for 20 minutes at room temperature, followed by PBS washes. Next, cells were stained with Giemsa (Sigma, USA) for 15 minutes at room temperature in the dark. Excess stain was removed, and the inserts were washed twice with PBS. Non-invasive cells were then removed from the upper chamber using cotton swabs. Finally, the number of invasive cells present on the other side of the insert was counted using a bright-field microscope.

### Breast tumor samples

Breast tumor along with adjacent normal tissues were obtained from American Oncology citizens Hospital-Hyderabad abiding by the norms of the institutional ethics committee -University of Hyderabad (UH/IEC/2015/86) as per the laid guidelines and protocols along with informed patient consent.

### Insilico analysis

The expression pattern of RUNX2 in normal and tumor breast tissue was analyzed using the GEPIA (http://gepia.cancer-pku.cn/detail.php?gene=&clicktag=expdiy) and UALCAN (https://ualcan.path.uab.edu/cgi-bin/ualcan-res.pl) web servers. Nodal metastasis status in breast cancer patients was assessed via the UALCAN database (https://ualcan.path.uab.edu/cgi-bin/ualcan-res.pl). To determine the tissue-specific expression of RUNX2, data from the Human Protein Atlas (https://www.proteinatlas.org/search/RUNX2) was utilized. Furthermore, overall survival (OS) and disease-free survival (DFS) analyses in breast cancer patients were performed using GEPIA (http://gepia.cancer-pku.cn/detail.php?gene=&clicktag=expdiy) to evaluate the prognostic relevance of RUNX2 expression.

### Mice xenograft and bone metastasis model

Six-week-old female FOX N1 athymic nude mice (n=4) were procured from CCMB animal house in India and acclimated in individually ventilated cages (IVCs) for one week under specific pathogen-free conditions at NIAB, India. All procedures involving animal studies were conducted in compliance with the ethical standards set by the Institutional Ethics Review Committee (IAEC) and approval (UH/IAEC/BMR/2021-1/04/R1. Intravenous injections of 1 x 10^5^ cells, including GFP-tagged EV, RUNX2 WT, RUNX2 S118A, and RUNX2 S118D mutant cell lines diluted in 100μl of PBS, were administered into the tail vein of each mouse, with four mice in each experimental group. Following a period of 6 weeks, animals were euthanized by CO2 inhalation followed by cervical dislocation to ensure death, and GFP intensity was assessed via immunoblotting. Tumor samples, bone marrow flush, and tissues from various organs were collected for analysis.

### Hematoxylin and eosin staining of tissue sections

Formalin-fixed, paraffin-embedded (FFPE) tissue samples were sectioned at a thickness of 4 μm using a microtome. The sections were mounted onto glass slides and subjected to deparaffinization in xylene, followed by rehydration through a graded series of ethanol (100%, 95%, 70%). Subsequently, the slides were stained with hematoxylin, rinsed in running tap water, and differentiated in acid alcohol. Following a brief bluing step, sections were counterstained with 1% eosin for 90 seconds. After dehydration through an ascending ethanol series and a final clearing step in xylene, slides were mounted using Cytoseal™ mounting medium and cover-slipped. Stained sections were visualized using a Leica trinocular microscope.

### Statistical analysis

All data points are represented as mean ± SEM. Statistical analysis was performed using GraphPad Prism software. Statistical significance was determined through a two-tailed unpaired Student’s t-test. For normally distributed data with multiple groups used one-way ANOVA. A P-values <0.05 were considered statistically significant. A minimum set of three independent experiments was carried out for all the cell line data and for tumor samples experiments were done in duplicates due to limited tissue availability.

## Results

### RUNX2 is a substrate of AMPK in breast cancer cells

**Figure 1:**
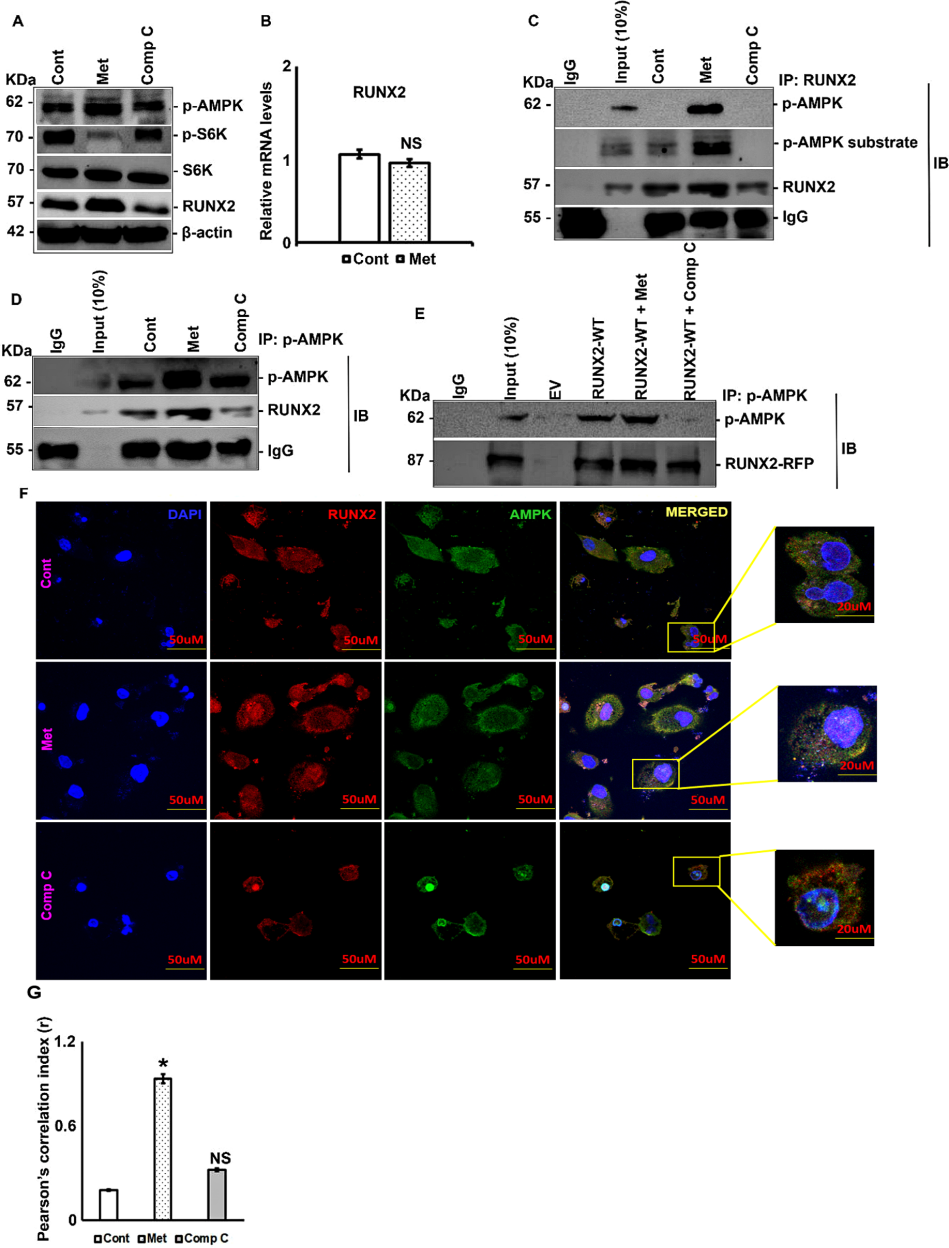
RUNX2 is a substrate of AMPK in breast cancer cells. MDA-MB-231 cells were treated with either metformin (20mM) or compound C (5μM) for 6 hours or none and subjected to **(A)** Western blot analysis and **(B)** RT-PCR analysis. MDA-MB-231 cells were treated with either metformin (20mM) or compound C (5μM) for 6 hours or none and subjected to IP analysis by **(C)** RUNX2 pull down and levels of RUNX2, p-AMPK substrate and p-AMPK were analyzed or by **(D)** p-AMPK pull down and levels of RUNX2, p-AMPK were analyzed. **(E)** MCF-7 cells were transfected with either RUNX2 WT or RUNX2 S118A or RUNX2 S118D or none, along with or without treatment of metformin (20mM) and Compound C (5μM) for 6 hours post to 48 hours of transfection and subjected to IP analysis by p-AMPK pull down and levels of p-AMPK and RUNX2-RFP were analyzed. **(F)** MDA-MB-231cells were treated with metformin (20mM) or compound C for 6 hours and subjected to immunofluorescence by anti-RUNX2 (Alexa 594) and anti-AMPK (Alexa 488) antibodies, counterstained with DAPI. **(G)** Immunofluorescence data were quantified to assess the degree of colocalization between AMPK and RUNX2 using ImageJ software. Mean ± S.E.M.; N = 3. *p<0.1 versus control, ^NS^p>0.1 versus control. The immunofluorescence and quantification experiments were carried out on three independent fields. The uncropped blots are provided in the **Supplementary Materials**. Cont, control; Met, metformin; Comp C, compound C; IP, immunoprecipitation; IB, immunoblotting; EV, empty vector; WT, wild type; NS, non-significant.

MDA-MB-231 cells were treated with metformin and compound C, following which the levels of RUNX2 were analyzed by immunoblotting and RT-PCR. Upon treatment with metformin, we observed an increase in RUNX2 protein levels (**Figure 1A**, **Supplementary Figure 1**). However, mRNA levels remained unaffected (**Figure 1B**). Notably, this increase in RUNX2 protein levels was reversed upon treatment with compound C (**Figure 1A**), an inhibitor of AMPK kinase activity. Subsequently, we investigated whether the interactions between AMPK and RUNX2 were sustained in MDA-MB-231 cells. For this purpose, RUNX2 was subjected to IP in MDA-MB-231 cells and immunoblotted with p-AMPK and p-AMPK substrate-specific antibodies. Metformin treatment enhanced AMPK and RUNX2 interaction (**Figure 1C**). To validate this interaction, IP was performed by p-AMPK, followed by immunoblotting for RUNX2. The results indicated that metformin treatment enhanced the interaction between RUNX2 and p-AMPK, which was lost when the cells were treated with compound C (**Figure 1D**). To confirm interactions between RUNX2 and AMPK, RUNX2 was overexpressed in MCF-7, and IP was carried out in the presence and/or absence of AMPK activators and inhibitors. Consistent with the above results, an interaction between p-AMPK and RUNX2 was observed following metformin treatment and was abolished upon treatment with compound C (**Figure 1E**). Immunofluorescence analysis was performed to confirm and quantify the interaction of p-AMPK and RUNX2. The physical interaction between RUNX2 and p-AMPK was evident upon metformin treatment (**Figures 1F, G**), further supporting the findings from immunoblotting and IP assays.

### AMPK-mediated phosphorylation of RUNX2 results in increased nuclear localization and transcriptional activity of RUNX2

**Figure 2:**
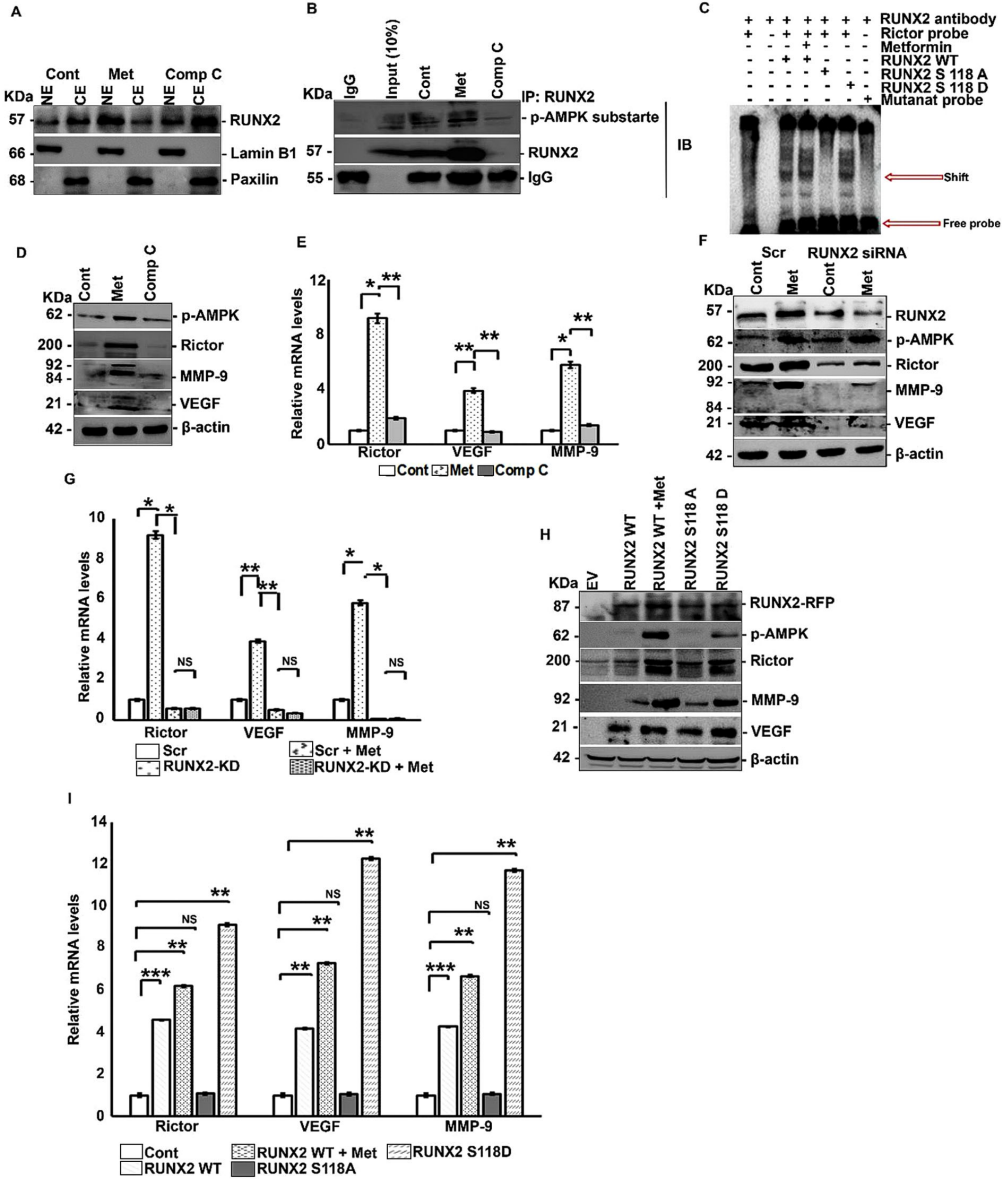
AMPK mediated phosphorylation of RUNX2 results in increased nuclear localization and transcriptional activity of RUNX2. MDA-MB-231 cells were treated with either metformin (20mM) or compound C (5μM) for 6 hours or none and subjected to nuclear- cytoplasmic extraction followed by **(A)** Western blot analysis and **(B)** IP analysis by RUNX2 pull down and levels of RUNX2, p-AMPK substrate and p-AMPK were analyzed. **(C)** MDA-MB-231 cells were treated with either metformin (20mM) or compound C (5μM) for 6 hours or none and nuclear extracts were subjected to EMSA. MDA-MB-231 cells were treated with either metformin (20mM) or compound C (5μM) for 6 hours and subjected to **(D)** Western blot analysis and **(E)** RT-PCR analysis. MDA-MB-231 cells were transfected with RUNX2 siRNA with or without metformin (20mM) treatment for 6 hours and subjected to **(F)** Western blot analysis and **(G)** RT-PCR analysis. MCF-7 cells were transfected with either RUNX2 WT or RUNX2 S118A or RUNX2 S118D or none, along with or without metformin (20mM) treatment for 6 hours post to 48 hours of transfection and subjected to **(H)** Western blot analysis and **(I)** RT-PCR analysis. Mean ± S.E.M.; N = 3. *p<0.1 versus control, **p<0.01 versus control, ***p<0.001 versus control, ^NS^p>0.1 versus control. The immunofluorescence and quantification experiments were carried out on three independent fields. The uncropped blots are provided in the **Supplementary Materials**. Cont, control; met, metformin; Comp C, compound C; CE, cytoplasmic extract; NE, nuclear extract; RUNX2-KD, RUNX2 knock down by siRNA; Scr, scrambled; EV, empty vector; WT, wild type; RUNX2A, RUNX2 S118A; RUNX2D, RUNX2 S118D; NS, non-significant.

Since RUNX2 is a transcription factor and AMPK phosphorylates RUNX2 in the DNA binding domain, we analyzed the effect of AMPK-induced RUNX2 phosphorylation on nuclear localization and function of RUNX2. To study the nuclear localization of RUNX2, cytoplasmic and nuclear fractionation was performed following treatment with metformin and compound C. Metformin treatment enhanced nuclear localization of RUNX2, which was reversed by compound C (**Figure 2A**). To confirm the role of RUNX2 phosphorylation in enhanced nuclear localization, we performed IP of RUNX2 in nuclear extracts and detected high levels of phospho-RUNX2 in the nuclear fraction upon metformin treatment (**Figure 2B**). Analysis of RUNX2 transcriptional targets implicated in breast cancer metastasis revealed that RUNX2 regulates the mTOR promoter (42). We also found that RUNX2 regulates mTORC2 (mammalian target of rapamycin complex 2) although the exact mechanism remains unclear (34). Bioinformatic analysis of the promoter of RICTOR, a key mTORC2 component, revealed the presence of RUNX2 a consensus-binding site (ACCACA, which is known as Osteoblast Specific cis acting Element2 (OSE2)) at -1884 kb. To confirm the role of RUNX2 Ser 118 phosphorylation, we transfected MCF-7 cells with RUNX2 wild type (WT), RUNX2 S118A (phosphonull mutant) and RUNX2 S118D (phosphomimic mutant) variants and subjected nuclear extracts to EMSA. RUNX2 WT treated with metformin and phosphomimic mutant exhibited potent binding to the RICTOR promoter compared to RUNX2 WT or phosphonull mutant (**Figure 2C**). Next, we evaluated the effect of RUNX2 phosphorylation on its transcriptional activity by measuring the mRNA and protein levels of its target genes, including RICTOR, VEGF, and MMP-9. MDA-MB-231 cells were treated with Metformin and/or compound C and protein and mRNA levels of RICTOR, VEGF (43) and MMP-9 (44) were assessed. Metformin upregulated RICTOR, MMP-9 and VEGF at both the protein and mRNA levels, while compound C suppressed their expression (**Figures 2D, E**).

To confirm the role of RUNX2 in metformin-mediated upregulation of RICTOR, VEGF and MMP-9, we performed knockdown of RUNX2 in MDA-MB-231 cells and measured RICTOR, VEGF and MMP-9 levels. RUNX2 knockdown blocked the upregulation of these genes, both in the presence and absence of metformin, indicating that RUNX2 is required for metformin-mediated upregulation of RICTOR, VEGF and MMP-9 (**Figures 2F, G**). To validate the role of Ser 118 phosphorylation in RUNX2-mediated upregulation of RICTOR, VEGF and MMP-9, we expressed RUNX2 WT, S118A & S118D in MCF-7 cells and evaluated target expression levels. We observed an upregulation of RUNX2 in metformin treated WT and phosphomimic mutant. This was reversed by transfection with phosphonull mutant or WT in the absence of metformin treatment (**Figures 2H, I**). These findings clearly indicate the role of RUNX2 Ser 118 phosphorylation in the upregulation of RICTOR, VEGF and MMP-9.

### mTORC2 is crucial for AMPK/RUNX2 axis

**Figure 3:**
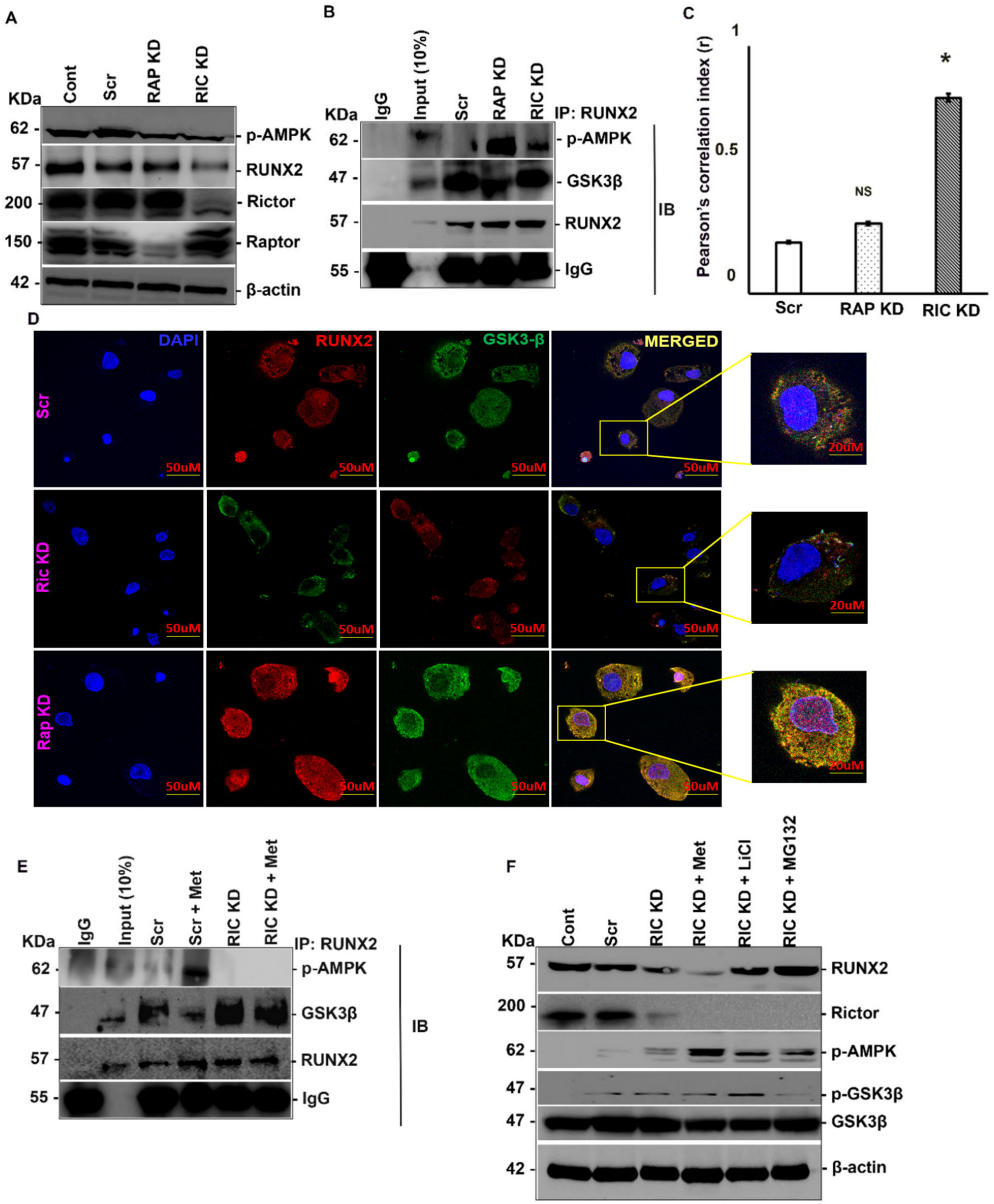
mTORC2 is crucial for AMPK/RUNX2 axis. MDA-MB-231 cells were transfected with siRNA’s against *RICTOR* and *RAPTOR* or none and 48 hours post transfection subjected to **(A)** Western blot analysis and **(B)** IP analysis by RUNX2 pull down and levels of RUNX2, GSK3β and p-AMPK were analyzed. MDA-MB-231 cells were transfected with siRNA’s against *RICTOR* and *RAPTOR* or none and 48 hours post transfection subjected to **(C)** Immunofluorescence data were quantified to assess the degree of colocalization between RUNX2 and GSK3β using ImageJ software. **(D)** immunofluorescence by anti-RUNX2 (Alexa 594) and anti-GSK3β (Alexa 488) antibodies counterstained with DAPI. **(E)** MDA-MB-231 cells were transfected with *RICTOR* siRNA with or without metformin (20mM) treatment for 6 hours and subjected to IP by RUNX2 pull down and levels of RUNX2, GSK3β and p-AMPK were analyzed. **(F)** MDA-MB-231 cells were transfected with *RICTOR* siRNA with or without metformin (20mM) or LiCl (0.5M) or MG-132 (3mM) treatment for 6 hours and subjected to Western blot analysis. Mean ± S.E.M.; N = 3. *p<0.1 versus scrambled, ^NS^p>0.1 versus scrambled. The immunofluorescence and quantification experiments were carried out on three independent fields. The uncropped blots are provided in the **Supplementary Materials**. Cont, control; Met, metformin; Comp C, compound C; IP, immunoprecipitation; IB, immunoblotting; Scr, scrambled; RAP, RAPTOR; RIC, RICTOR; NS, non-significant.

RICTOR, which is upregulated by metformin treatment in a RUNX2-dependent manner, is a key component required for mTORC2 activation (45). mTORC2 is involved in RUNX2 regulation through the inhibition of GSK3β (34), a downstream target of mTORC2 involved in RUNX2 repression. GSK3β can also phosphorylate RUNX2 in the runt domain, exerting an inhibitory effect on RUNX2 activity (38). We analyzed the effect of the p-AMPK/RUNX2 axis on the interaction between RUNX2 and GSK3β. To elucidate the role of mTORC2 in RUNX2 regulation, RICTOR and RAPTOR, key components of mTORC2 &C1 respectively, were knocked down by siRNA treatment, followed by analysis of RUNX2 levels. The downregulation of RUNX2 was specific to RICTOR knockdown only (**Figure 3A**, **Supplementary Figure 2**), indicating that mTORC2 plays a critical role in maintaining RUNX2 stability and functions upstream of AMPK in this pathway.

To validate role of mTORC2 in RUNX2 regulation, we performed RUNX2 IP following RICTOR knock down and analyzed its interaction with p-AMPK and GSK3β. RICTOR knockdown abolished the interaction between RUNX2 and p-AMPK while enhancing the interaction between RUNX2 and GSK3β (**Figure 3B**). Immunofluorescence confirmed the interaction between GSK3β and RUNX2 in the presence or absence of RICTOR (**Figures 3C, D**). To elucidate the effect of mTORC2 on RUNX2 and p-AMPK interaction, we performed IP with RUNX2 after RICTOR knockdown in the presence or absence of metformin. Metformin treatment had no effect on RUNX2’s interaction with GSK3β in the absence of RICTOR. Similarly, upregulated p-AMPK was unable to bind to RUNX2 in the absence of RICTOR. In the presence of RICTOR, this situation was reversed (**Figure 3E**). To confirm that GSK3β mediates mTORC2 action, cells were treated with LiCl, an inhibitor of GSK3 kinase activity, metformin and MG-132 along with RICTOR downregulation. The downregulation of RUNX2 induced by RICTOR knockdown was rescued upon treatment with either LiCl or with MG-132, but not in response to metformin treatment (**Figure 3F**). This highlights the significance of mTORC2 as an upstream regulator of the AMPK/RUNX2 axis, indicating its crucial role in mediating the interaction between AMPK and RUNX2.

### Metformin promotes EMT and induces osteoblast like phenotype to breast cancer cells through p-AMPK/RUNX2/mTORC2 axis

**Figure 4:**
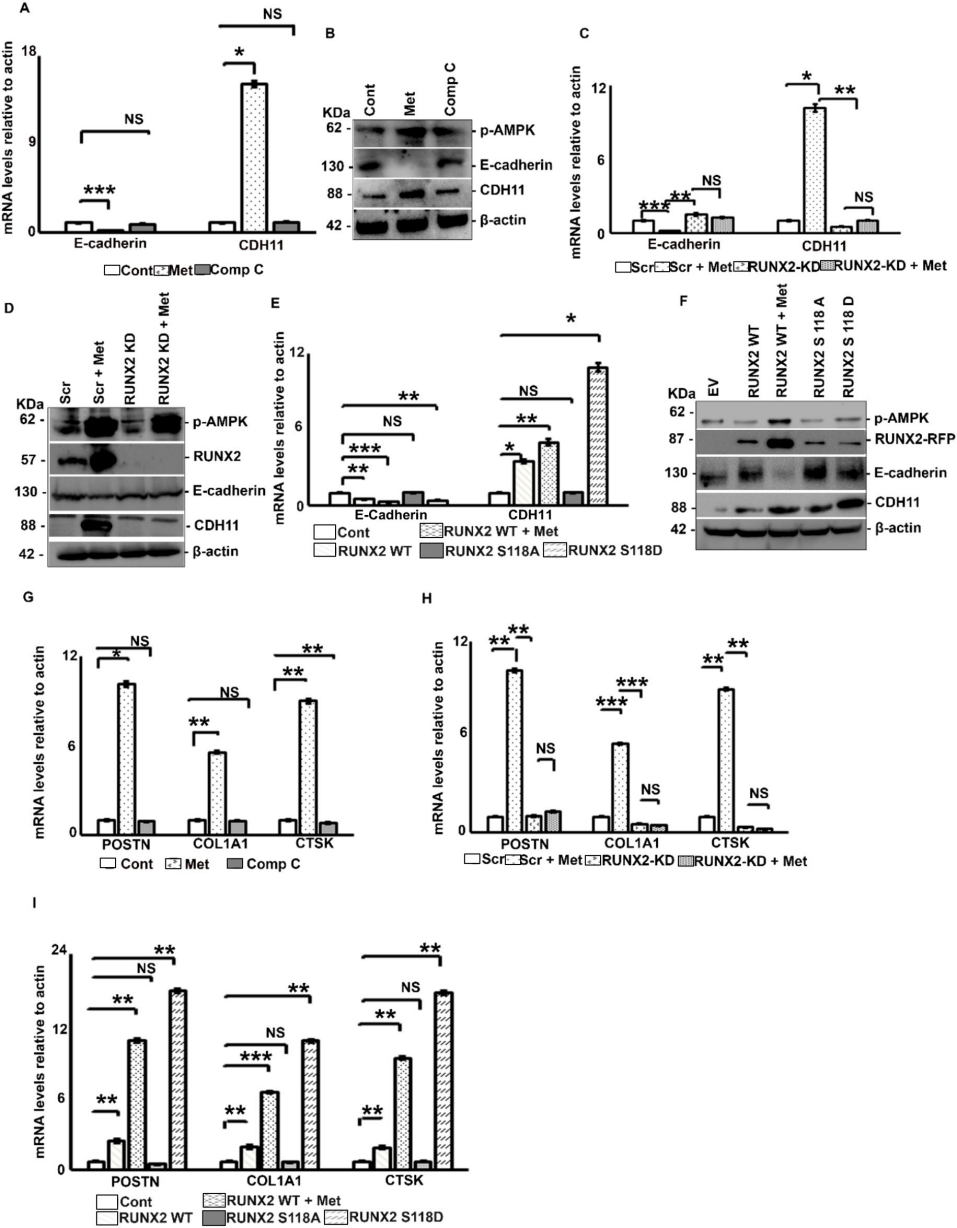
Metformin promotes EMT and induces osteoblast like phenotype to breast cancer cells through p-AMPK/RUNX2/mTORC2 axis. MDA-MB-231 cells were treated with either metformin (20mM) or compound C (5μM) for 6 hours or none and subjected to **(A)** RT-PCR analysis and **(B)** Western blot analysis. MDA-MB-231 cells were transfected with siRNA against RUNX2 or none, and 48 hours post-transfection with or without metformin (20mM) treatment for 6 hours, and subjected to **(C)** RT-PCR analysis and **(D)** Western blot analysis. MCF-7 cells were transfected with either RUNX2 WT or RUNX2 S118A or RUNX2 S118D or none, along with or without metformin (20mM) treatment for 6 hours post to 48 hours of transfection and subjected to **(E)** RT-PCR analysis and **(F)** Western blot analysis. **(G)** MDA-MB-231 cells were treated with either metformin (20mM) or compound C (5μM) for 6 hours or none and subjected to RT-PCR analysis. **(H)** MDA-MB-231 cells were transfected with siRNA against RUNX2 or none and 48 hours post-transfection with or without metformin (20mM) treatment for 6 hours and subjected to RT-PCR analysis. **(I)** MCF-7 cells were transfected with either RUNX2 WT or RUNX2 S118A or RUNX2 S118D or none, along with or without metformin (20mM) treatment for 6 hours post to 48 hours of transfection and subjected to RT-PCR analysis. Mean ± S.E.M.; N = 3. *p<0.1 versus scrambled or control, **p<0.01 versus scrambled or control, ***p<0.001 versus scrambled or control, ^NS^p>0.1 versus scrambled or control. The immunofluorescence and quantification experiments were carried out on three independent fields. The uncropped blots are provided in the **Supplementary Materials**. Cont, control; met, metformin; Comp C, compound C; IP, immunoprecipitation; IB, immunoblotting; Scr, scrambled; RUNX2KD, knock down of RUNX2 using siRNA; WT, wild type; RUNX2A, RUNX2 S118A; RUNX2D, RUNX2 S118D; NS, non-significant; POSTN, periostin; CTSK, cathepsin K; COL1A1, type I collagen.

The initial step in EMT induction involves the transcriptional repression of E-cadherin. Upon treatment with metformin, MDA-MB-231 exhibited transcriptional repression of E-cadherin in a RUNX2-dependent manner, as illustrated in **Figures 4A, B**. To validate the impact of S118 phosphorylation in E-cadherin suppression, MCF-7 cells were transfected with phospho-mutants, and E-cadherin levels were assessed with or without metformin treatment. Notably, cells transfected with RUNX2 WT exhibited elevated E-cadherin suppression, a phenomenon further enhanced by metformin treatment and the presence of RUNX2 S118D mutant (**Figure 4F**). The next important step in EMT is the expression of appropriate mesenchymal markers such as CDH11 (46). In MDA-MB-231 cells, CDH11 levels increased post-metformin treatment and decreased upon RUNX2 knockdown (**Figures 4C, D**), indicating the role of RUNX2 in CDH11 upregulation. This relationship between CDH11 upregulation and RUNX2 Ser 118 phosphorylation was confirmed by transfecting MCF-7 cells with RUNX2 variants. Transfection with RUNX2 resulted in upregulation of CDH11, which was reversed by transfection with the phosphonull mutant (**Figures 4E, F**). Lastly, for cancer cells to home in bones, they must express bone-specific surface markers like CDH11, ECM proteins such as type I collagen (COL1A1), periostin (POSTN), cathepsin K (CTSK) and bone remodeling transcription factors (like RUNX2). In bone metastasizing breast cancer patients, nearly 57 BRG’s were expressed (42). To investigate if metformin can induce an osteoblast-like phenotype in breast cancer cells, we treated MDA-MB-231 cells with metformin and its inhibitor compound C and analyzed the expression of COL1A1, POSTN and CTSK by RT-PCR. Results demonstrated that three genes were upregulated in metformin-treated samples, with this upregulation being lost upon pre-treatment with compound C (**Figure 4G**). Furthermore, the upregulation of COL1A1, POSTN and CTSK mediated by metformin was dependent on RUNX2 expression, as evidenced by the loss of expression following RUNX2 knock down, even in the presence of metformin (**Figures 4H, E**). Additionally, this upregulation was also dependent on Ser 118 phosphorylation, as observed in MCF-7 cells transfected with RUNX2 variants (**Figure 4I**).

### Metformin promotes chemotaxis/metastasis of transformed breast cancer cells

**Figure 5:**
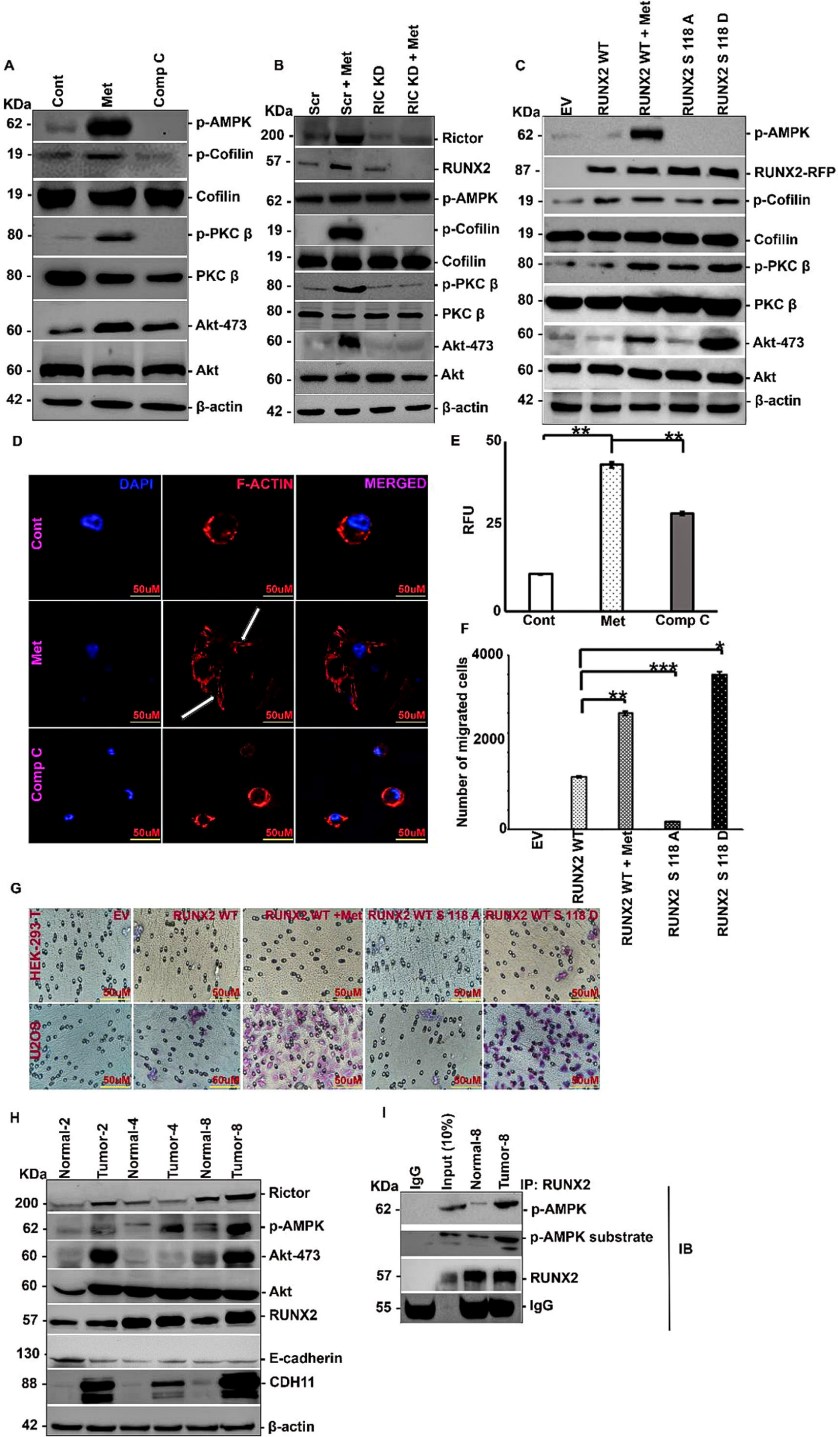
Metformin promotes chemotaxis/metastasis of transformed breast cancer cells. **(A)** MDA-MB-231 cells were treated with either metformin (20mM) or compound C (5μM) for 6 hours or none and subjected to Western blot analysis. **(B)** MDA-MB-231 cells were transfected with siRNA against *RICTOR* or none and 48 hours post transfection with or without metformin (20mM) treatment for 6 hours and subjected to Western blot analysis. **(C)** MCF-7 cells were transfected with either RUNX2 WT or RUNX2 S118A or RUNX2 S118D or none, along with or without metformin (20mM) treatment for 6 hours post to 48 hours of transfection and subjected to Western blot analysis. **(D)** MDA-MB-231 cells were treated with either metformin (20mM) or compound C (5μM) for 6 hours or none and subjected to immunofluorescence stained using Rhodamine-phalloidin (540), counter stained by DAPI. **(E)** Quantification of fluorescence signal using ImageJ. **(F)** Quantification of number of migrated cells by electron microscopy. **(G)** MCF-7 cells were transfected with either RUNX2 WT or RUNX2 S118A or RUNX2 S118D or none, along with or without metformin (20mM) treatment for 6 hours post to 48 hours of transfection and subjected to migration through collagen coated membrane, with lower chambers coated with either HEK-293T cells or U2OS cells. **(H)** Breast tumor tissue along with adjacent normal tissue were subjected to protein isolation followed by Western blot analysis and **(I)** IP by RUNX2 pull down and levels of p-AMPK, RUNX2 and p-AMPK substrate-specific motif were analyzed. Mean ± S.E.M.; N = 3. *p<0.1 versus control or WT, ^NS^p>0.1 versus control. The immunofluorescence and quantification experiments were carried out on three independent fields. The uncropped blots are provided in the **Supplementary Materials**. Cont, control; Met, metformin; Comp C, compound C; IP, immunoprecipitation; IB, immunoblotting; Scr, scrambled; EV, empty vector; WT, wild type. **p<0.01 versus control or WT, ***p<0.001 versus control or WT.

The subsequent step in cancer progression to EMT involves metastasis/cell motility. Activation of metastasis requires the digestion of ECM proteins by upregulation of MMPs. Another major event is actin reorganization to aid in cell movement (47). mTORC2 is the major regulator of cytoskeleton organization (48). Previous research has shown that metformin treatment can upregulate mTORC2 through RICTOR. Based on these findings, we analyze the downstream effectors of mTORC2: PKCs and AKT are well known targets of mTORC2 that are involved in Rac1 activation, leading to cofilin phosphorylation and actin severing (29). Metformin treatment activated PKCβ and AKT through phosphorylation, resulting in cofilin phosphorylation (**Figure 5A**) in a RUNX2 and mTORC2-dependent manner (**Figure 5B**). To confirm the involvement of the RUNX2/AMPK axis in actin reorganization, we transfected MCF-7 cells with RUNX2 Ser 118 phosphorylation variants and observed similar result (**Figure 5C**). The formation of actin stress fibers is a prerequisite for cell migration (49). Metformin treatment resulted in the formation of F-actin stress fibers in MDA-MB-231 cells, reversed by pre-treatment with compound C (**Figure 5D, E**). The formation of stress fibers leads to chemotaxis of cells towards a favorable secondary niche, such as bone in this case. We next evaluated the role of AMPK-induced RUNX2 Ser 118 phosphorylation on bone specific movement of MCF-7 cells transfected with RUNX2 WT or S118A or S118D with or without metformin treatment. MCF-7 cells transfected with RUNX2 WT, coupled with metformin treatment and phosphomimic mutant, had a higher proportion of migrated cells compared to RUNX2 WT alone or phosphonull mutant (**Figure 5F**). Migration of MCF-7 cells was repressed when the lower chamber was seeded with HEK-293T cells, indicating that AMPK-induced RUNX2 Ser 118 phosphorylation-mediated chemotaxis is specific to the bone microenvironment (**Figure 5G**).

### RUNX2 expression correlates with clinical phenotype in breast tumor tissues

To investigate the potential role of RUNX2 in breast cancer, we have retrieved the data from publicly available datasets and performed in-silico analysis. In-silico analysis revealed a significant clinical correlation of RUNX2 expression with breast cancer progression. A pan-cancer expression profile using TCGA data (**Supplementary Figure 3A**) revealed elevated RUNX2 transcript levels in various tumor types, with a marked overexpression in breast cancer (BRCA) samples compared to normal tissues. Further analysis using the GEPIA and UALCAN portals (**Supplementary Figures 3B, C**) confirmed significantly higher expression of RUNX2 in primary breast tumor tissues compared to normal breast tissues. In addition, we explored the correlation of RUNX2 expression with nodal metastasis status in breast cancer patients (**Supplementary Figure 3D**). The results indicated a progressive increase in RUNX2 expression from node-negative (N0) to node positive (N1-N3) stages, suggesting a potential role in metastatic progression. Tissue specific expression of RUNX2 was validated using immunohistochemistry data from the Human Protein Atlas (**Supplementary Figure 3E**). Representative Immunohistochemistry (IHC) images of breast cancer subtypes, including lobular and ductal carcinoma, demonstrated moderate to strong nuclear localization of RUNX2 protein in tumor tissues. To access the prognostic relevance of RUNX2, we performed Kaplan-Meier survival analysis using GEPIA. Patients with high RUNX2 expression exhibited reduced OS and DFS compared to those with low RUNX2 expression (**Supplementary Figures 3F, G**). We validated our *in silico* and *in vitro* data by examining tumor tissue and adjacent normal tissue samples and observed increased levels of p-AMPK and RUNX2 in tumor samples compared to normal samples. Consistent with the upregulation of RUNX2, the downstream targets RICTOR and AKT-473 also showed increased expression in tumor samples. Additionally, tumors exhibited increased expression of CDH11 and decreased expression of E-cadherin compared to normal samples (**Figures 5H**, **Supplementary Figure 4A**). To assess RUNX2 Ser 118 phosphorylation by AMPK, we performed IP pull-down of RUNX2 in both normal and tumor tissues, followed by IB with p-AMPK and p-AMPK substrate-specific antibody. We found that, in tumor samples, RUNX2 and p-AMPK interactions remained intact and that RUNX2 was phosphorylated, as indicated by the presence of p-AMPK substrate-specific antibody signals (**Figure 5I**, **Supplementary Figures 4B, C**).

### RUNX2 promotes bone metastasis

**Figure 6:**
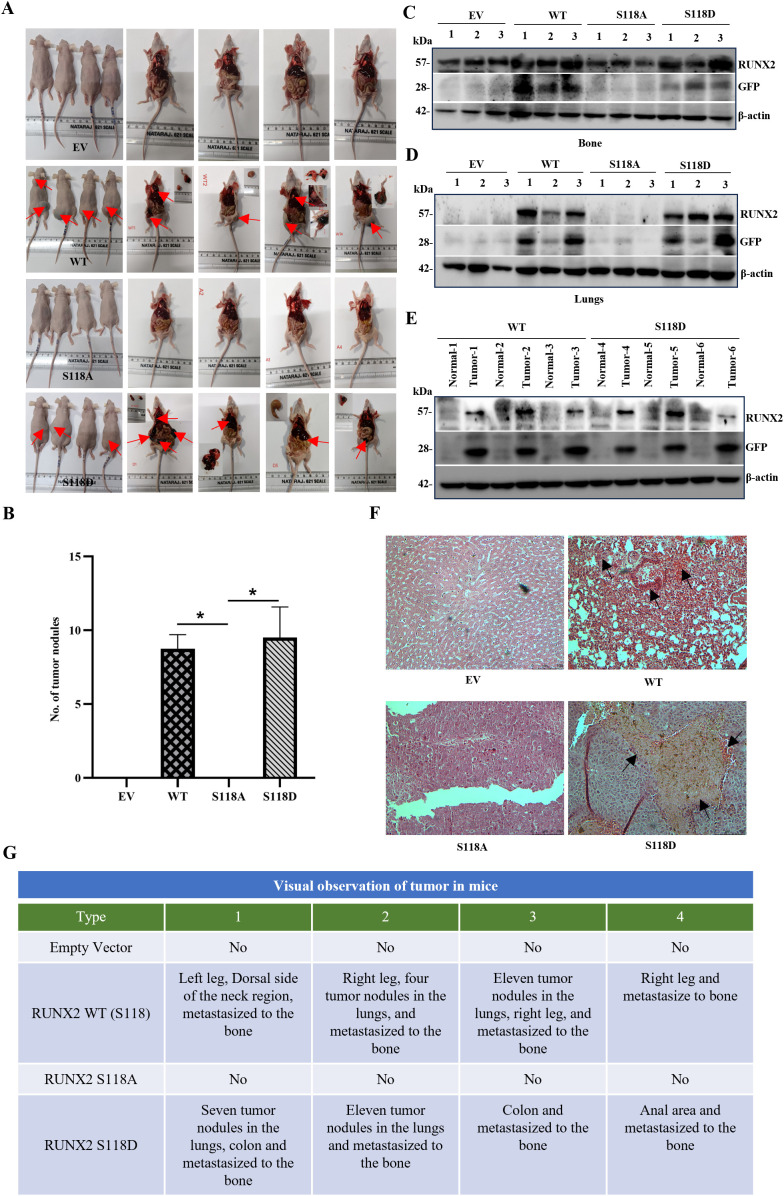
RUNX2-Dependent Bone Metastatic Outgrowth of MCF-7 Cells in NOD-SCID Mice. This figure illustrates the experimental setup and outcomes of intravenous injections of MCF-7 cell lines with distinct RUNX2 expressions, emphasizing the impact on bone metastatic outgrowth in NOD-SCID mice (n=4 per group). **(A)** Intravenous Injection and Tumor Growth Observation. MCF-7 cell lines overexpressing RUNX2 WT, RUNX2 S118A, and RUNX2 S118D mutants were intravenously injected into the tail vein of NOD-SCID mice. Metastatic outgrowth was visually monitored for tumor growth. **(B)** Quantification of tumornodules observed in the lungs of WT and mutant phenotypes. **(C)** Bone Marrow Cell Collection and GFP Intensity Assessment. Bone marrow cells were obtained through bone flush, and the GFP intensity was assessed through immunoblot analysis. **(D)** Lung tissue protein isolation and GFP intensity Check. GFP intensity was evaluated by isolating proteins from lung tissues and subjecting them to immunoblot analysis. **(E)** Tumor and normal tissue collection for GFP intensity analysis. Tumors, along with adjacent normal tissues, were collected. Proteins were isolated, and GFP intensity was checked through immunoblot analysis. **(F)** Histopathological examination by hematoxylin and eosin staining of lung tissues **(G)** Visual observation table. The table provides a visual summary of tumor observations and lung metastatic nodules in different groups of NOD-SCID mice. The uncropped blots are provided in the **Supplementary Materials**. EV, Empty vector; WT, Wild type.

To elucidate the involvement of RUNX2 in bone metastasis, we conducted experiments using six-week-old female FOX N1 athymic nude mice (n=4). We established MCF-7 cell lines overexpressing RUNX2 WT, RUNX2 S118A, and RUNX2 S118D (**Supplementary Figure 5**). We then used these stable cell lines to establish bone metastasis models through intravenous injection. After a 6-week duration, our *in vivo* data revealed that MCF-7 cell lines overexpressing RUNX2 WT and RUNX2 S118D exhibited multiple tumors at various sites, particularly in the legs (**Figure 6A**) and tumor nodules in the lungs (**Figure 6B**). This phenotype was reversed in MCF-7 cell lines overexpressing RUNX2 S118A, where both metastasis and tumor formation were notably reduced (**Figures 6A–E**). To access the histological impact of RUNX2 overexpression and its mutant subtypes, H & E staining was performed on lung tissues from all four experimental groups. Tumors from the EV group exhibited moderately differentiated morphology with low nuclear pleomorphism and sparse mitotic activity, consistent with a less aggressive phenotype. In contrast both RUNX2 WT and S118D expressing groups displayed increased nuclear atypia, and denser cellular architecture, suggestive of enhanced proliferative and possibly invasive potential. The S118A mutant, however showed morphological features closely resembling with the EV, indicating reduced tumor aggressiveness in the absence of Ser 118 phosphorylation (**Figure 6F**). Consistent with these observations, MCF-7 cell lines overexpressing high levels of RUNX2 WT and RUNX2 S118D demonstrated increased migration toward the lungs and bone compared to MCF-7 cell lines overexpressing RUNX2 S118A (**Figure 6G**). These findings underscore the critical role of RUNX2 phosphorylation and stability in the development of bone metastasis in nude mice.

## Disscussion

Breast cancer survival rates vary widely, ranging from nearly 80% in early-stage disease to below 40% in advanced stages, where aggressive phenotypes often exhibit enhanced metastatic potential and therapeutic resistance (50). Among metastatic sites, the bone remains the most frequent and clinically challenging, contributing significantly to disease recurrence and drug resistance (51).

Metformin, a widely prescribed biguanide for type 2 diabetes, has recently emerged as a potential anti-cancer agent (52, 53). Its antitumor effects have largely been attributed to the activation of AMPK and subsequent inhibition of mTORC1, a critical regulator of cellular proliferation and protein synthesis (54). Nevertheless, the influence of metformin on EMT and metastatic progression remains insufficiently defined, with reports suggesting context-dependent outcomes. Our previous study demonstrated that metformin exerts osteoprotective effects under diabetic conditions through AMPK-dependent phosphorylation and stabilization of RUNX2 at Ser118 (40). Given the central role of RUNX2 in tumor progression and metastasis, we investigated whether metformin could modulate the stability and activity of RUNX2 in breast cancer cells. Interestingly, we observed a sustained interaction between p-AMPK and RUNX2 in both triple-negative and hormone receptor-positive breast cancer cell lines (MDA-MB-231 and MCF-7), suggesting that AMPK-mediated RUNX2 stabilization is maintained in the context of cancer. AMPK phosphorylation within the runt homology domain appears to enhance RUNX2’s DNA-binding capacity and nuclear localization, aligning with its role as a transcriptional activator of metastasis-associated genes (27). RUNX2 upregulation has been long associated with the acquisition of invasive and premetastatic traits in multiple cancers (55, 56). Consistent with this, metformin-treated cells displayed increased expression of key RUNX2 targets, such as MMP-9, VEGF, and RICTOR factors, which are known to promote ECM degradation, angiogenesis, and cytoskeletal remodeling during EMT, as reported in (43, 57).

Furthermore, our findings indicate that GSK3β-mediated phosphorylation negatively regulates RUNX2 stability, whereas mTORC2-via its core component RICTOR-acts as an upstream inhibitor of GSK3β (38, 58). Loss of RICTOR enhanced the RUNX2-GSK3β interaction and decreased RUNX2 levels, effects that were rescued by pharmacological inhibition of GSK3β, underscoring a feed-forward relationship between RUNX2 and mTORC2. Notably, these results suggest that the mTORC2 complex may function as a critical node linking AMPK activation to the stabilization of RUNX2. Bone metastasis is a hallmark of aggressive breast cancer and often requires tumor cells to acquire osteoblast-like characteristics to colonize the bone microenvironment (8, 24, 59). In our study, metformin-mediated stabilization of RUNX2 correlated with the expression of osteogenic markers, suggesting that under a specific molecular context, metformin can influence metastatic tropism toward the bone.

Collectively, our findings reveal an unexpected role of the AMPK–mTORC2–AKT–RUNX2 signaling axis in regulating breast cancer progression. While metformin’s canonical effects are tumor-suppressive through mTORC1 inhibition, our data reveal that, in certain molecular settings characterized by high RUNX2 expression, AMPK activation can paradoxically enhance pathways linked to EMT and bone metastasis (Figure 7). This highlights the importance of cellular context and oncogenic background in determining the therapeutic outcome of metformin. We propose that RUNX2 expression levels could serve as a potential biomarker to predict differential responses to metformin in breast cancer patients. Future studies should validate these findings in preclinical and clinical settings to better define the dualistic nature of metformin’s actions in cancer and to guide its safer and more effective therapeutic application.

## Data Availability

The original contributions presented in the study are included in the article/**Supplementary Material**. Further inquiries can be directed to the corresponding author.

## Supplementary material

Supplementary Figure 1 MDA-MB-231 cells were treated with either metformin (20mM) or compound C (5μM) for 6 hours or none, and have a quantitative plot of p-AMPK, p-S6K, S6K and RUNX2 normalized to β-actin. Mean ± S.E.M.; N = 3. *p<0.1 versus control, **p<0.01 versus control, ***p<0.001 versus control, ^NS^p>0.1 versus control.

Supplementary Figure 2 MDA-MB-231 cells were transfected with siRNAs against RICTOR and RAPTOR or none, and 48 hours post-transfection and a quantitative plot of p-AMPK, RUNX2, RICTOR and RAPTOR normalized to β-actin. Mean ± S.E.M.; N = 3. *p<0.1 versus control, **p<0.01 versus control, ***p<0.001 versus control, ^NS^p>0.1 versus control.

Supplementary Figure 3 Insilico analysis of RUNX2 expression and clinical correlation in breast cancer. A. Pan-cancer analysis of RUNX2 expression across various tumor and normal tissues from the TCGA database. B & C. RUNX2 expression levels in normal versus primary breast tumor samples. D. RUNX2 expression in breast cancer patients stratified by nodal metastasis status. E. Tissue-specific expression of RUNX2 based on immunohistochemistry (IHC) analysis from the Human Protein Atlas, showing staining in different subtypes of breast carcinoma, including lobular and ductal carcinoma. Kaplan–Meier plot of F. OS and G. DFS comparing patients with high versus low RUNX2 expression levels.

Supplementary Figure 4 Breast tumor tissue along with adjacent normal tissues were subjected to protein isolation followed by **(A)** Western blot analysis and **(B, C)** IP by RUNX2 pull down and levels of p-AMPK, RUNX2 and p-AMPK substrate-specific motif were analyzed.

Supplementary Figure 5 RUNX2 in EMT Induction and Metastasis in MCF-7 Cell Lines. The impact of RUNX2 on EMT and metastasis in MCF-7 cell lines. Overexpression of both RUNX2 WT and the phospho-mimetic mutant RUNX2 S118D resulted in a notable promotion of the mesenchymal marker N-cadherin, accompanied by a reduction in E-cadherin expression. Conversely, MCF-7 cell lines overexpressing the empty vector (EV) and the phospho-null mutant RUNX2 S118A exhibited an increase in E-cadherin expression along with a concurrent decrease in N-cadherin expression.
