# Supplementary material for: AMPK/mTORC2/AKT-473/RUNX2 signaling axis modulates epithelial-mesenchymal transition and bone tropism in breast cancer
Source: Front Oncol. 2026 Apr 10;16:1785903. doi: 10.3389/fonc.2026.1785903 (PMC13105891; doi:10.3389/fonc.2026.1785903)
Supplement: Supplementary file 1 [file Table1.docx]

**Supplementary Table 1:** Primers used in the PCR

| **Primers** | **Sequence (5’ – 3’)** |
| --- | --- |
| RUNX2 WT (FP) | CCGCTCGAGACCATGGCATCAAACAGCCTCTTCAGC |
| RUNX2 WT (RP) | GGAAGATCTCGATATGGTCGCCAAACAGATTCATC |
| RUNX2 S118 A(FP) | GTCCGCACCGACGCCCCCAACTTCCTG |
| RUNX2 S118 A(RP) | CAGGAAGTTGGGGGCGTCGGTGCGGAC |
| RUNX2 S118 D(FP) | CGCACCGACGACCCCAACTTC |
| RUNX2 S118 D(RP) | GAAGTTGGGGTCGTCGGTGCG |
