# Supplementary material for: AMPK/mTORC2/AKT-473/RUNX2 signaling axis modulates epithelial-mesenchymal transition and bone tropism in breast cancer
Source: Front Oncol. 2026 Apr 10;16:1785903. doi: 10.3389/fonc.2026.1785903 (PMC13105891; doi:10.3389/fonc.2026.1785903)
Supplement: Supplementary file 2 [file Table2.docx]

**Supplementary Table 2:** Primers used in the RT PCR

| **Primers** | **Sequence (5’ – 3’)** |
| --- | --- |
| RICTOR (FP) | GGAAGCCTGTTGATGGTGAT |
| RAPTOR (RP) | GGCAGCCTGTTTGGTGT |
| VEGF (FP) | AGTTAGTCACACTGGAGATTGAC |
| VEGF (RP) | ATAGGATCGACAGTTGTAACC |
| MMP-9 (FP) | ATAGACTACTACAGGCT |
| MMP-9 (RP) | TAGCACGGATAGACCA |
| RUNX2 (FP) | CTCTGCACCAAGTCCTTTTAATC |
| RUNX2 (RP) | AGGAGGGGTAAGACTGGTCATAG |
| Periostin (FP) | AATGGAAGGAATGAAAGGCTG |
| Periostin (RP) | CCTCGATCTCCTCCCTCAGT |
| CDH11 (FP) | AGACGTTGGATCGAGAAGAG |
| CDH11 (RP) | GGTTGTCCTTCGAGGATACT |
| Cathepsin K (FP) | TCCATCCATAACCTTGAGGCTT |
| Cathepsin K (RP) | CCACAGCCATCATTCTCAGACACA |
| Collagen Type 1 Alpha 1 (FP) | TCTGCGACAACGGCAAGGTG |
| Collagen Type 1 Alpha 1 (RP) | GACGCCGGTGGTTTCTTGGT |
