# Supplementary figures and images for: AMPK/mTORC2/AKT-473/RUNX2 signaling axis modulates epithelial-mesenchymal transition and bone tropism in breast cancer
Source: Front Oncol. 2026 Apr 10;16:1785903. doi: 10.3389/fonc.2026.1785903 (PMC13105891; doi:10.3389/fonc.2026.1785903)

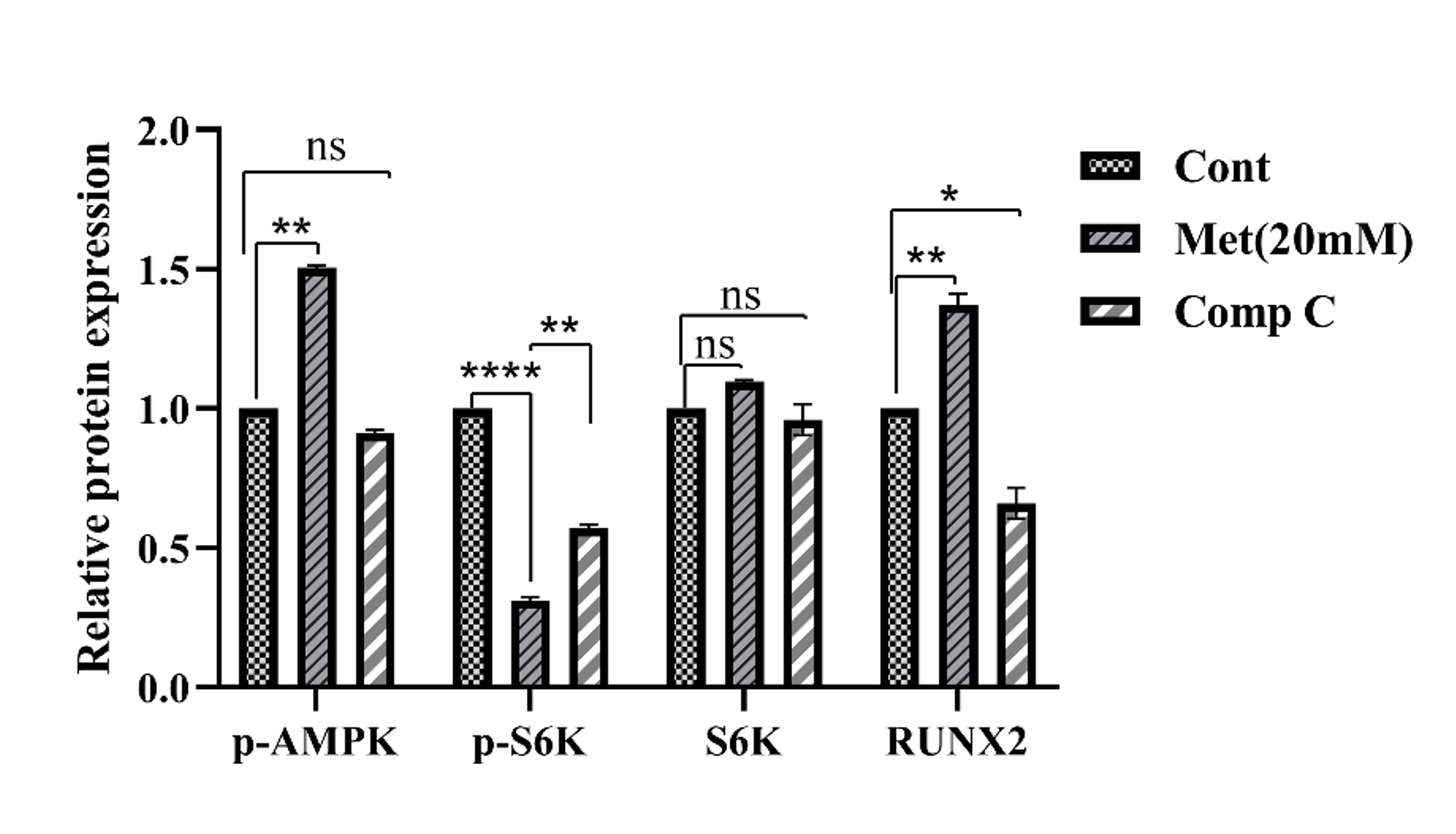

Supplement: Supplementary Figure 1 — MDA-MB-231 cells were treated with either metformin (20mM) or compound C (5μM) for 6 hours or none, and have a quantitative plot of p-AMPK, p-S6K, S6K and RUNX2 normalized to β-actin. Mean ± S.E.M.; N = 3. *p<0.1 versus control, **p<0.01 versus control, ***p<0.001 versus control, NSp>0.1 versus control. [file Image1.tif]

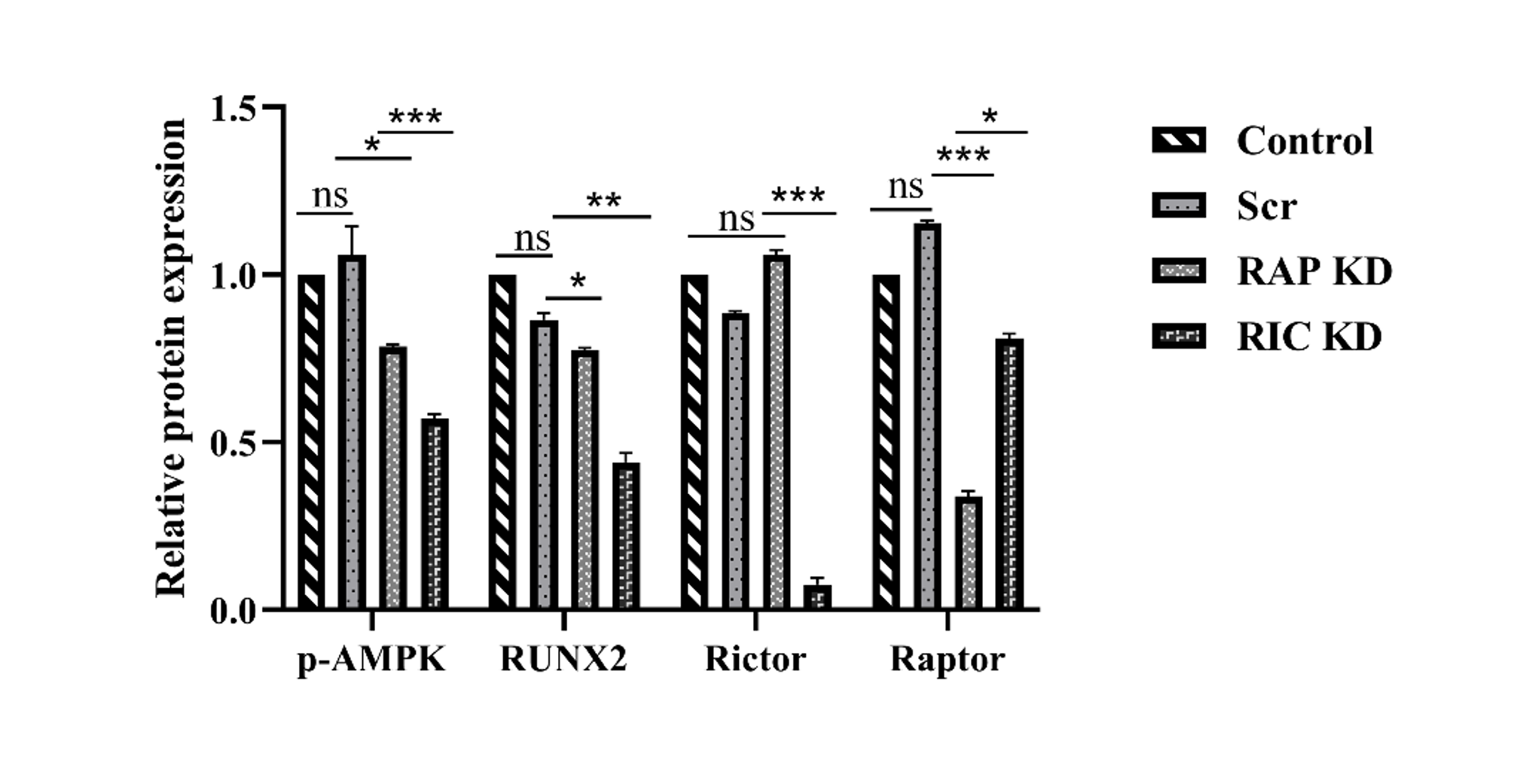

Supplement: Supplementary Figure 2 — MDA-MB-231 cells were transfected with siRNAs against RICTOR and RAPTOR or none, and 48 hours post-transfection and a quantitative plot of p-AMPK, RUNX2, RICTOR and RAPTOR normalized to β-actin. Mean ± S.E.M.; N = 3. *p<0.1 versus control, **p<0.01 versus control, ***p<0.001 versus control, NSp>0.1 versus control. [file Image2.tif]

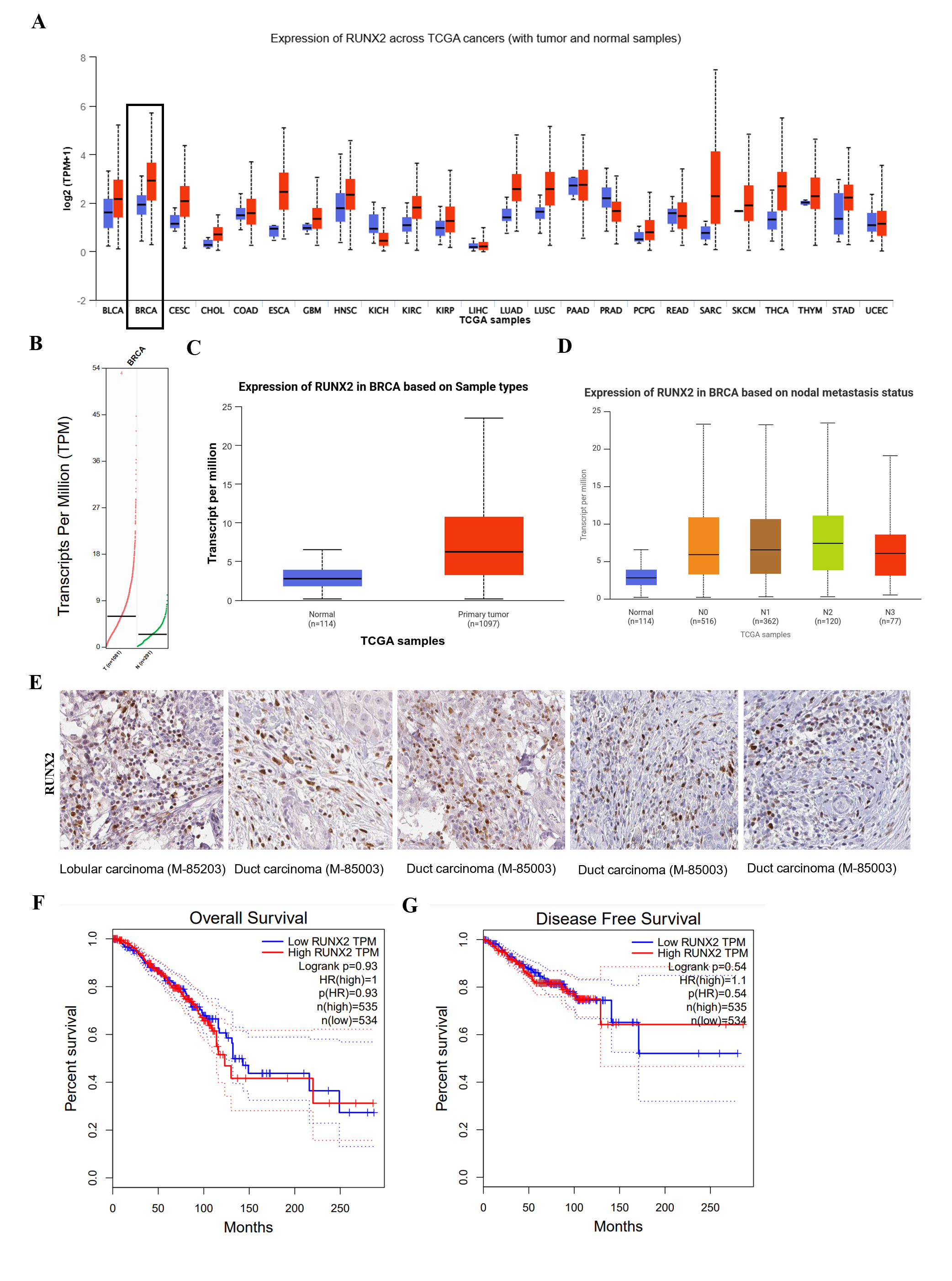

Supplement: Supplementary Figure 3 — Insilico analysis of RUNX2 expression and clinical correlation in breast cancer. A. Pan-cancer analysis of RUNX2 expression across various tumor and normal tissues from the TCGA database. B & C. RUNX2 expression levels in normal versus primary breast tumor samples. D. RUNX2 expression in breast cancer patients stratified by nodal metastasis status. E. Tissue-specific expression of RUNX2 based on immunohistochemistry (IHC) analysis from the Human Protein Atlas, showing staining in different subtypes of breast carcinoma, including lobular and ductal carcinoma. Kaplan–Meier plot of F. OS and G. DFS comparing patients with high versus low RUNX2 expression levels. [file Image3.tif]

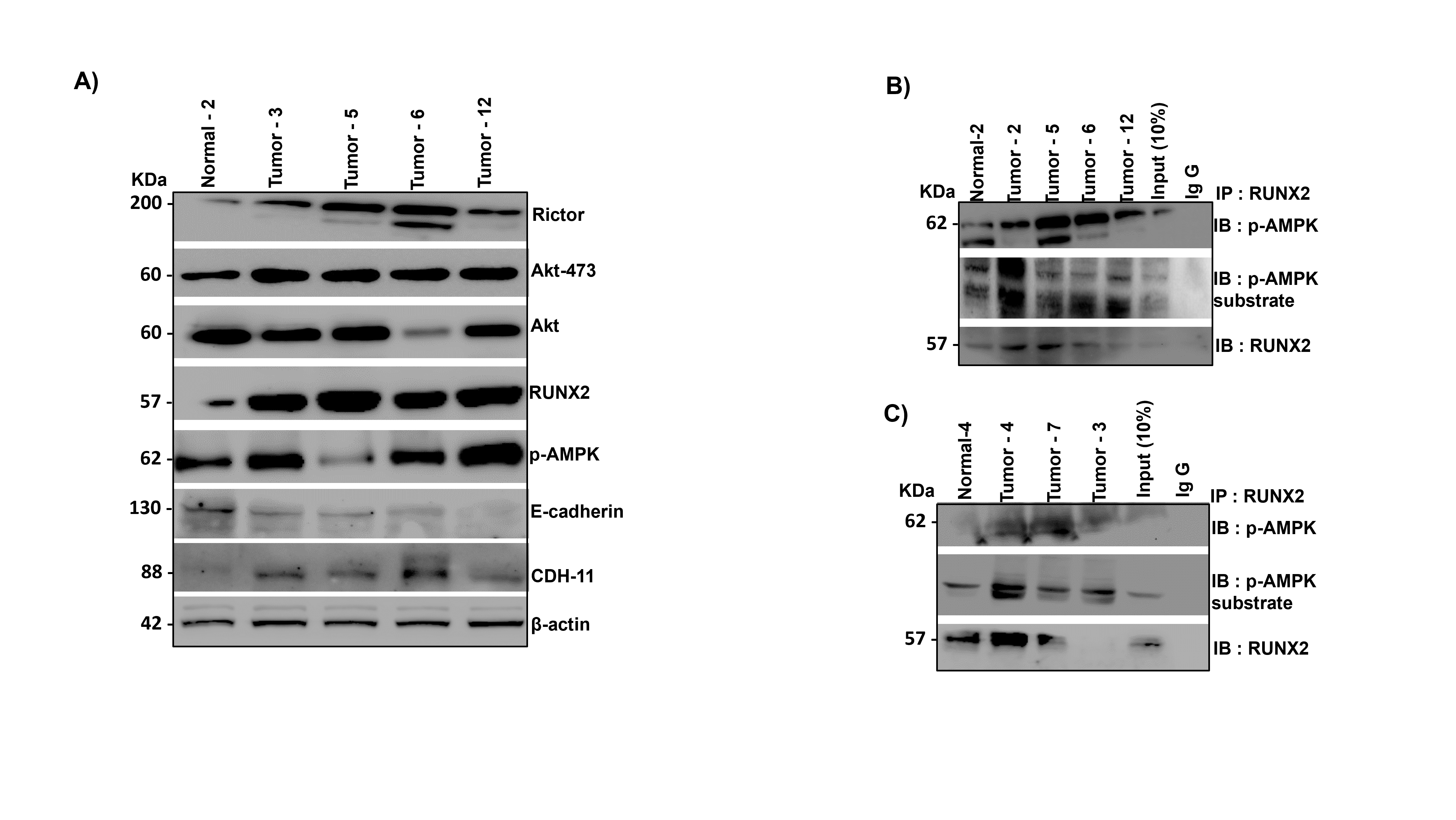

Supplement: Supplementary Figure 4 — Breast tumor tissue along with adjacent normal tissues were subjected to protein isolation followed by (A) Western blot analysis and (B, C) IP by RUNX2 pull down and levels of p-AMPK, RUNX2 and p-AMPK substrate-specific motif were analyzed. [file Image4.tif]

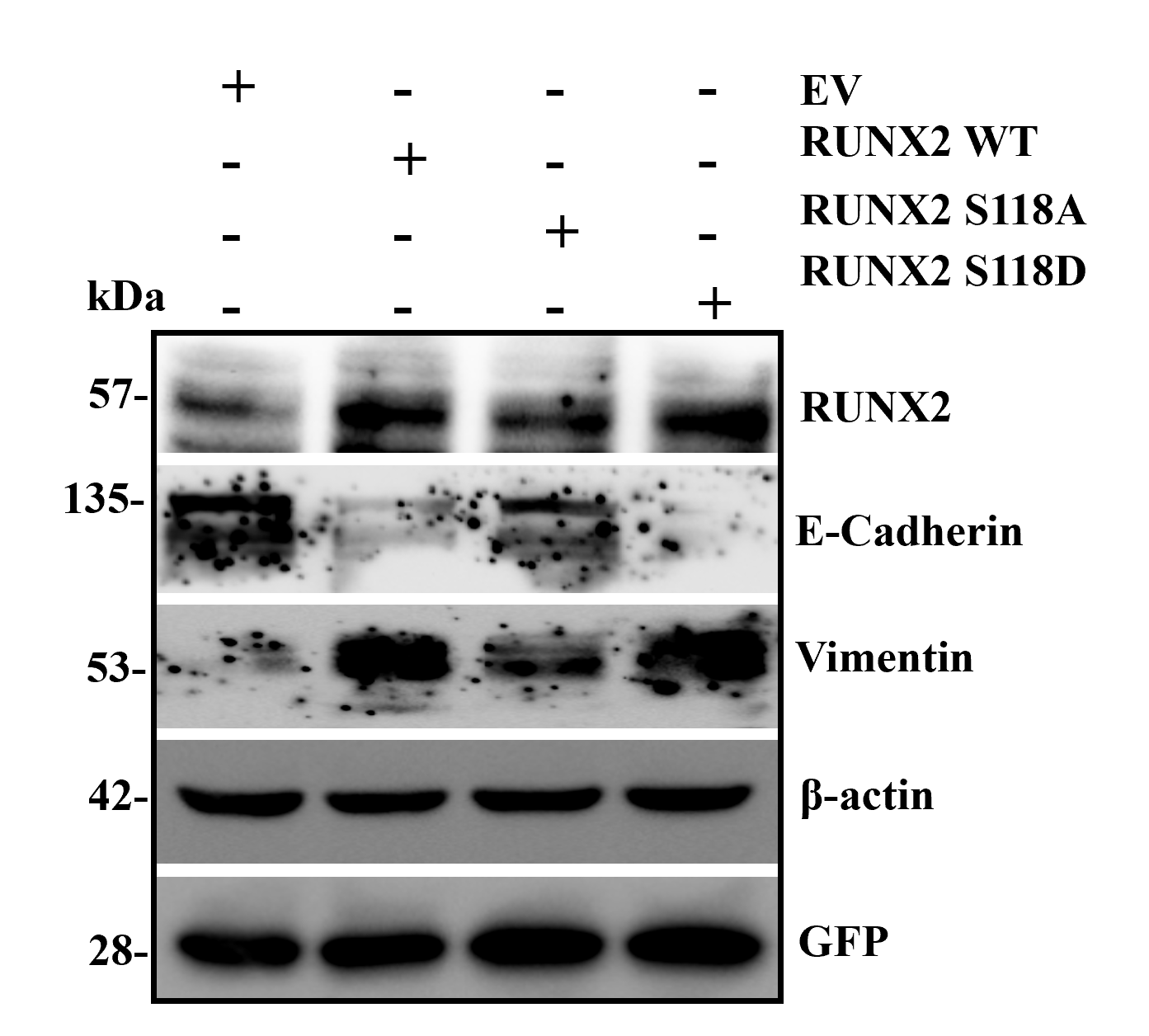

Supplement: Supplementary Figure 5 — RUNX2 in EMT Induction and Metastasis in MCF-7 Cell Lines. The impact of RUNX2 on EMT and metastasis in MCF-7 cell lines. Overexpression of both RUNX2 WT and the phospho-mimetic mutant RUNX2 S118D resulted in a notable promotion of the mesenchymal marker N-cadherin, accompanied by a reduction in E-cadherin expression. Conversely, MCF-7 cell lines overexpressing the empty vector (EV) and the phospho-null mutant RUNX2 S118A exhibited an increase in E-cadherin expression along with a concurrent decrease in N-cadherin expression. [file Image5.tif]
